# Intraobserver and interobserver reproducibility of M-mode and B-mode acquired mitral annular plane systolic excursion (MAPSE) and its dependency on echocardiographic image quality in children

**DOI:** 10.1371/journal.pone.0196614

**Published:** 2018-05-10

**Authors:** Kai O. Hensel, Markus Roskopf, Lucia Wilke, Andreas Heusch

**Affiliations:** 1 HELIOS University Medical Center Wuppertal, Children’s Hospital, Department of Pediatrics, Center for Clinical & Translational Research (CCTR), Faculty of Health, Center for Biomedical Education & Research (ZBAF), Witten/Herdecke University, Faculty of Health, Wuppertal, Germany; 2 University of Cambridge, Addenbrooke’s University Hospital, Department of Pediatrics, Cambridge, United Kingdom; Kurume University School of Medicine, JAPAN

## Abstract

**Background:**

Mitral annular plane systolic excursion (MAPSE) is an increasingly used echocardiography technique to assess left ventricular (LV) function. However, reproducibility and dependence on echocardiographic image quality for MAPSE in pediatric patients have not been studied to date.

**Methods:**

We analyzed 284 transthoracic echocardiograms performed on consecutive normotensive children without structural heart disease (mean age 12.6±3.1 years, 50.4% female). B-mode and M-mode derived MAPSE measurements were performed and analyzed regarding inter- and intraobserver reliability and the influence of echocardiographic image quality.

**Results:**

Overall, MAPSE measurements were highly reproducible with only minor bias. Both inter- and intraobserver reliability were significantly better for M-mode derived MAPSE (p<0.001). Echocardiographic image quality did not significantly influence M-mode MAPSE reproducibility (p>0.235). In contrast, B-mode lateral MAPSE was significantly better reproducible in optimal image quality (-0.07±1.04) when compared to suboptimal echocardiographic images (0.42±1.59, p<0.001). Moreover, poor quality images yielded significantly lower M-mode MAPSE values (14.3±2 mm) than near-optimal (15.2±1.9 mm, p<0.001) or optimal images (15.1±2.2 mm, p = 0.006).

**Conclusion:**

Echocardiographic image quality essentially has a negligible effect on MAPSE reproducibility and measurements. Consequently, MAPSE is a robust echocardiographic parameter with convincing reproducibility for the assessment of LV function in children—even in patients with substandard imaging conditions.

## Background

The assessment of left ventricular (LV) function is among the most common indications for transthoracic echocardiography. Several methods for the quantification of LV performance are out there, but no single one of them is perfect. Since the 1960s, the echocardiographic determination of LV stroke volume is frequently performed [1]. Mostly, LV ejection fraction (EF) is used to express LV performance. This can be achieved by numerous methods, all of which are prone to several limitations: the need of echocardiographic expertise, reader variability and discordance, the technical quality of the echocardiographic study and exact endocardial image resolution [2].

Mitral annular plane systolic excursion (MAPSE), synonymous for atrioventricular displacement, descent of the base or mitral annular motion, is another echocardiographic method for the determination of LV function. It was first described as a phenomenon in 1967 [3] and as a useful diagnostic technique in the late 1980s [4]. MAPSE measures LV longitudinal shortening, which has been established as a sensitive parameter reflecting and the primary contributor to facilitate LV pump function [5]. Subsequently, several studies have demonstrated its significance and feasibility as a promising surrogate parameter for LV function in various clinical settings and patient populations. MAPSE reflected LV deterioration in patients with heart failure and preserved EF [6], in adult males with severely impaired LV EF [7] and in critically ill patients with shock [8]. Moreover, MAPSE detection was found to be feasible, an easier technique, and shorter in duration compared with the eyeball method in the determination of LV EF in mechanically ventilated obese patients [9]. Diastolic function can also be assessed with it, i.e. in obese adults with normal LV EF [10]. Furthermore, even when performed by an untrained observer MAPSE measurements were found to be a highly accurate predictor of EF [11]. Other studies reported good correlations of MAPSE and other LV assessment methodologies such as three-dimensional echocardiography or magnetic resonance imaging [12]. Recently, a fully automatic algorithm for the detection of LV dysfunction based on MAPSE measurements was introduced [13]. Moreover, cardiac biomarkers like NT-proBNP, galectin-3 and high sensitivity troponin T and I reflect cardiac MRI derived MAPSE [14, 15]. Finally, MAPSE was shown to be a relevant prognostic index. In asymptomatic patients with aortic stenosis and normal EF, an asymptomatic decrease in MAPSE was associated with the clinical need for aortic valve intervention despite ongoing preservation of LVEF [16].

Only few studies have investigated the use of MAPSE in children. MAPSE Z-scores were proven useful for assessing global LV function in children with various body sizes [17]. For instance, in children with acute-phase Kawasaki disease MAPSE z-scores beyond a cutoff value of -0.9 served as an indicator to detect LV dysfunction [18]. Furthermore, MAPSE was shown to be depressed in children with pulmonary stenosis and restored after percutaneous balloon pulmonary valvuloplasty [19]. Another clinical implication is the early detection of ventricular dysfunction in pediatric oncology patients following anthracycline chemotherapy [20].

The beauty of utilizing MAPSE measurements lies in its simplicity. It can be easily performed, does not require dedicated training or longstanding echocardiographic expertise and is supposed to be less dependent on endocardial border resolution. However, MAPSE reliability in pediatric patients and the importance of image quality in children is still unknown. Therefore, the aim of this study was to investigate inter- and intraobserver reproducibility of MAPSE in children and to evaluate the significance of echocardiographic image quality.

## Methods

### Study population

In this study we analyzed 284 echocardiograms of consecutive normotensive children without structural heart disease between 6 and 17 years of age (mean age 12.6±3.1 years, 63 were younger than 10 years); 50.4% female. All study participants were recruited at the Children’s Hospital at Helios University Medical Centre Wuppertal, Germany either as healthy siblings of treated patients or as patients in good physical health. Inclusion criteria were unimpaired physical health, good fitness and the absence of cardiovascular disease. Primary exclusion criteria were the presence of any compromising features such as fatigue, pain, fever or other past or present health conditions currently affecting physical fitness or the cardiovascular system. This included but was not limited to congenital heart disease, heart failure, acquired valvular disease, kidney disease, developmental delay, obesity or pathologic ECG-changes. One patient was excluded from the study after initially having been enrolled as he was diagnosed with heart disease and 12 patients were excluded due to insufficient echocardiographic image quality (n = 12). A priori, we established a study design to categorize the study cohort according to either flawless, near-optimal or substandard echocardiographic image quality. All study participants underwent a thorough physical examination and clinical assessment according to standardized protocols by trained and certified staff. The study was carried out in accordance with the declaration of Helsinki’s ethical principles for medical research involving human subjects and approved by the Witten/Herdecke University ethics committee. For all study participants a written consent was signed by the child itself and by the legal guardian(s).

### Transthoracic echocardiography

**Fig 1:**
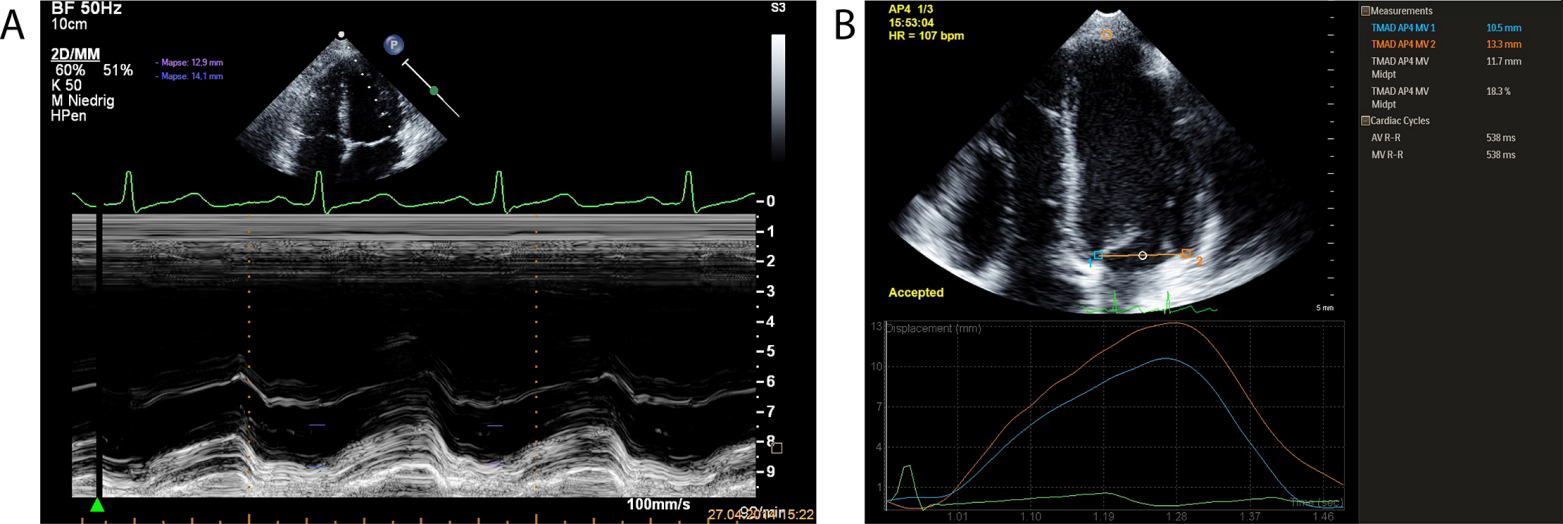
Echocardiographic image samples of MAPSE imaging. **A.** M-mode derived MAPSE measurement, **B.** B-mode derived MAPSE measurement.

All study participants underwent a thorough standard transthoracic echocardiography study according to recommendations of the American Society of Echocardiography [21]. All involved examiners were trained according to the guidelines and standards for performance of a pediatric echocardiogram as proposed by the Task Force of the Pediatric Council of the American Society of Echocardiography [22]. We used the commercially available ultrasound device iE33 by Phillips Ultrasound Inc., USA, with a S5-1 Sector Array transducer (Sector 1–5 MHz). All images were digitally recorded and subsequently transferred to an offline workstation for analysis, using XCelera Version 3.1.1.422 and QLAB Version 10 by Phillips Ultrasound Inc., USA. For speckle tracking derived strain and strain rate analyses images were digitally stored in DICOM format and transferred to an off-line workstation for postprocessing. Two-dimensional greyscale M-mode and B-mode images were recorded in standard parasternal short- and long-axis views as well as in apical 4-, 3- and 2-chamber views. Tissue harmonic imaging was used to enhance 2D image quality. Images were obtained at the level of the LV and the aortic valve to assess left atrial diameter, aortic root diameter, fractional shortening, LV cavity and LV posterior wall, interventricular septum, LV mass, LV enddiastolic and endsystolic volumes, EF and stroke volume. The modified Simpson’s biplane method was used to calculate EF. PW-Doppler and PW-Tissue-Doppler imaging was used to measure E/A-ratio, mitral deceleration time and E/E’-ratio for the assessment of LV diastolic function as previously described [23]. All echocardiographic measurements were evaluated utilizing pediatric specific Z-scores [24]. MAPSE was assessed in the 4-chamber view on the LV lateral and septal borders of the mitral annulus as well as utilizing M-mode at the LV lateral border of the mitral ring as previously described [25, 26] (Fig 1). Specifically, the distance between the nadirs of the annulus motion profile corresponding to the maximal backward motion of the mitral annulus from the apex after the P-wave to the maximal shortening defined as point of peak upward excursion was measured. Caution was paid to align the sample volume as vertical as feasible with regard to the heart apex. As recommended elsewhere, three consecutive cardiac cycles were assessed and averaged [27]. LV length was defined in the apical 4-chamber view as the distance from the mitral annular plane to the apical epicardium at end-diastole.

### Image quality scoring

Echocardiographic images were categorized according to echocardiographic image quality. In detail, LV myocardial wall visualization was analyzed by a trained echocardiographer and rated according to a previously published score raging from “0” to “3” [28]. A score of “0” was assigned to images without clear endocardial border delineation or absent samples. Images with <70% myocardial wall visualization were scored “1”, “2” was assigned when 70–95% of the relevant wall structures were visualized and “3” referred to images with > 95% endocardial border visualization.

### Assessment of reproducibility

Inter- and intraobserver variabilities were analyzed by additional evaluation of B-mode and M-Mode images by a second independent interpreter, who was blinded to the results of the first echocardiographic reader. To determine intraobserver (day-to-day) variability, one echocardiographic examiner repeated the MAPSE measurements in a random order with a minimum of 48 hours between two corresponding analyses.

### Biostatistical analyses

Epidemiological and clinical parameters, hemodynamics data, echocardiographic data and differences between echocardiographic measurements were presented as mean and standard deviation. The association between two continuous data was quantified by Spearman's rank correlation coefficient and illustrated by Scatterplots. To describe the reproducibility of MAPSE measurements the difference and the absolute difference between two measurements were calculated and described as mean, standard deviation, median, minimum and maximum. Inter- and intraobserver agreement was analyzed by calculating the 95%-confidence intervals of the mean difference between the two measurements and 95% limits of agreement (mean difference ± 1.96 x standard deviation (SD) of the difference). Differences between the measurements were illustrated by Bland-Altman plots. Whether the reproducibility of B-mode lateral was the same as that of B-mode septal and M-Mode was analyzed by comparing the absolute differences of these methods with the Wilcoxon signed-rank test for two related samples. Whether the reproducibility depends on image quality was tested by pairwise comparisons of the three image quality groups by Mann-Whitney-U-Test. All statistical tests were two-sided and p-values < 0.05 were considered statistically significant. Microsoft Windows Excel Version 16.0 and Stata/IC 14.2 for Windows (College Station, TX) were utilized for statistical analysis.

## Results

### Patient characteristics and conventional echocardiographic parameters

Baseline epidemiological and echocardiographic data is presented in Table 1. 284 echocardiographs from normotensive children and adolescents free of cardiovascular disease were included in this study. Mean age was 12.6±3.1 years and 50.4% were female. All epidemiological parameters including weight (50.1±17.4 kg), height (156.2±16.8 cm) and BMI (19.9±4.1) were normal as evaluated by Z-scores. Hemodynamic monitoring revealed normal heart rate (78±13 beats/minute), systolic (112±14.7 mmHg) and diastolic (67±12 mmHg) blood pressure.

**Table 1:** Baseline clinical characteristics and echocardiographic parameters derived from two-dimensional and Doppler imaging.

	Study group (n = 284)
**Age** (years)	12.6±3.1
**Female** (%)	50.4
**Height** (cm)	156.2±16.8
**Weight** (kg)	50.1±17.4
**Body surface** (m^2^)	1.5±0.3
**Body mass index** (kg/m^2^)	19.9±4.1
**Heart rate** (beats/minute)	78±13
**Blood pressure, systolic** (mmHg)	112±14.7
**Blood pressure, diastolic** (mmHg)	67±12
**LA/AoR**	1.1±0.16
**Fractional shortening (%)**	34.7±3.6
**Interventricular septal end-diastolic diameter** (cm)	0.9±0.2
**LV end-diastolic diameter** (cm)	4.2±0.5
**LV posterior wall diameter, diastolic** (cm)	0.9±0.3
**LV mass** (g)	112.6±41.3
**End-diastolic volume of the left ventricle** (ml)	95.3±36.6
**Ejection fraction** (%)	59±5.6
**Stroke volume** (ml)	51.7±20.1
**E-Wave / A-Wave**	1.8±0.4
**Mitral deceleration time** (s)	0.2±0.1
**E/E’** (cm/s)	7.5±2.4
**Global circumferential strain** (%)	-23.7±4.6
**MAPSE, M-mode lateral** (mm)	15±2
**MAPSE, B-mode lateral** (mm)	12.5±2.4
**MAPSE, B-mode septal** (mm)	12.3±2
**MAPSE / length**	0.22±0.05

Left atrium/aortic root ratio was 1.1±0.17, fractional shortening was 34.7±3.6%, EF was 59±5.6% and stroke volume was 51.7±20.1 ml. Diastolic function was unremarkable as detected by E/A wave of 1.8±0.4 mitral deceleration time of 0.2±0.1 seconds and E/E’ ratio of 7.5±2.4. Longitudinal and circumferential strain rate was assessed using speckle tracking imaging yielding normal parameters as compared to previously published reference values [29]. M-mode derived lateral MAPSE was 15±2 mm and B-mode lateral and septal MAPSE were 12.5±2.4 mm and 12.3±2 mm, respectively. Altogether, 101 single MAPSE measurements were lower than two SD below the age specific mean reference values [30]. Out of those, 96 MAPSE detections were lower than 2 SD below the age specific mean in only one of the applied modalities (i.e. low B-mode but normal M-mode derived MAPSE, etc.). 5 patients had MAPSE < 2SD of the age specific mean in all three MAPSE measurements. In these patients EF was normal. Correlations of MAPSE and other echocardiographic myocardial performance parameters are described in the supplemental material (S1 Text, S1 Table and S1 Fig).

### Inter- and intraobserver variability of MAPSE imaging

**Fig 2:**
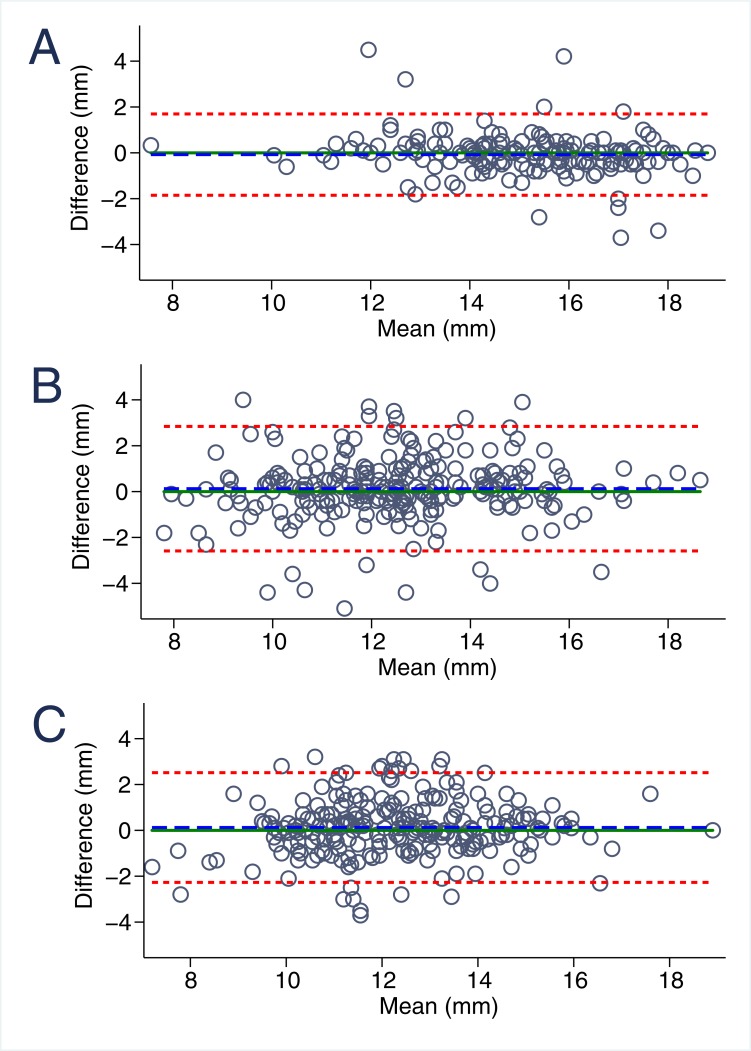
Bland-Altmann graphic: Reproducibility of MAPSE imaging. **A.** Interobserver variability for m-mode derived MAPSE, **B.** Interobserver variability for B-Mode lateral derived MAPSE, **C.** Interobserver variability for B-Mode septal derived MAPSE.

**Fig 3:**
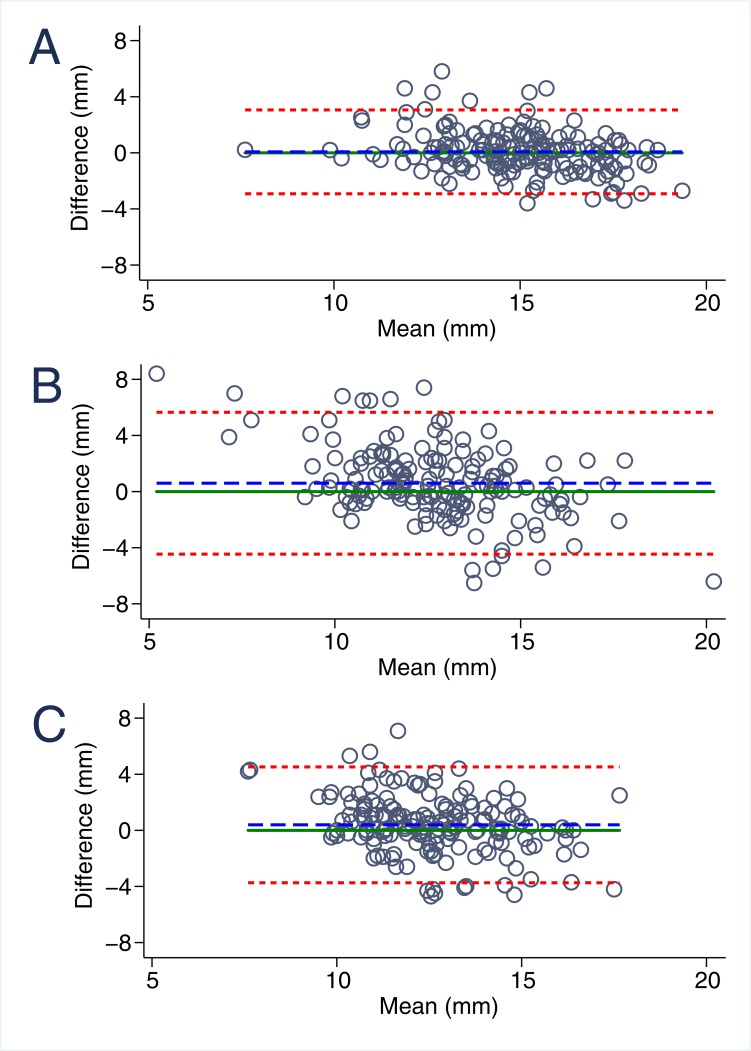
Bland-Altmann graphic: Reproducibility of MAPSE imaging. **A.** Intraobserver variability for m-mode derived MAPSE, **B.** Intraobserver variability for B-Mode lateral derived MAPSE, **C.** Intraobserver variability for B-Mode septal derived MAPSE.

Overall, MAPSE measurements were highly reproducible (Table 2, Figs 2 and 3). Mean differences in interobserver analyses were minor both for M-mode (-0.08±0.91 mm) as well as B-mode derived septal (0.12±1.22 mm) and lateral (0.13±1.39 mm) MAPSE. Intraobserver variability was similar in M-mode and slightly increased in B-mode MAPSE (Fig 3). For both inter- and intraobserver comparisons limits of agreement were narrowest in M-mode (-1.85; 1.70) followed by septal (-2.27; 2.52) and lateral (-2.59; 2.84) B-mode MAPSE. Accordingly, M-Mode MAPSE was significantly better reproducible than B-mode lateral MAPSE both for inter- (p<0.001) and intraobserver comparisons (p<0.001).

**Table 2:** Inter- and intraobserver reproducibility of b-mode and m-mode derived MAPSE measurements.

		B-mode		M-mode Lateral
		Septal	Lateral	
**INTEROBSERVER**				
	**Mean difference**	0.12±1.22	0.13±1.39	-0.08±0.91
	**Absolute difference**	0.90±0.83	0.96±1.0	0.54±0.73
	**Comparison with B-Mode lateral#**	p = 0.995	-	0.89
	rho*	0.80	0.79	0.89
	**95%-CI of mean difference**	-0.03; 0.28	-0.05; 0.30	-0.21; 0.05
	LOA**	-2.27; 2.52	-2.59; 2.84	-1.85;1.70
**INTRAOBSERVER**				
	**Mean difference**	0.39±2.11	0.60±2.58	0.07±1.53
	**Absolute difference**	1.60±1.42	1.92±1.82	1.12±1.04
	**Comparison with B-Mode lateral#**	p = 0.033	-	p<0.001
	rho*	0.52	0.52	0.75
	**95%-CI of mean difference**	0.07; 0.72	0.20; 1.00	-0.16; 0.29
	LOA**	-3.73; 4.52	-4.46; 5.65	-2.93; 3.06

# Wilcoxon signed-rank test

* Spearman's rank correlation coefficient

** LOA 95% limits of agreement (mean difference ± 1.96 standard deviation of the difference)

LAO = limits of agreement

### The impact of echocardiographic image quality on MAPSE reproducibility

**Fig 4:**
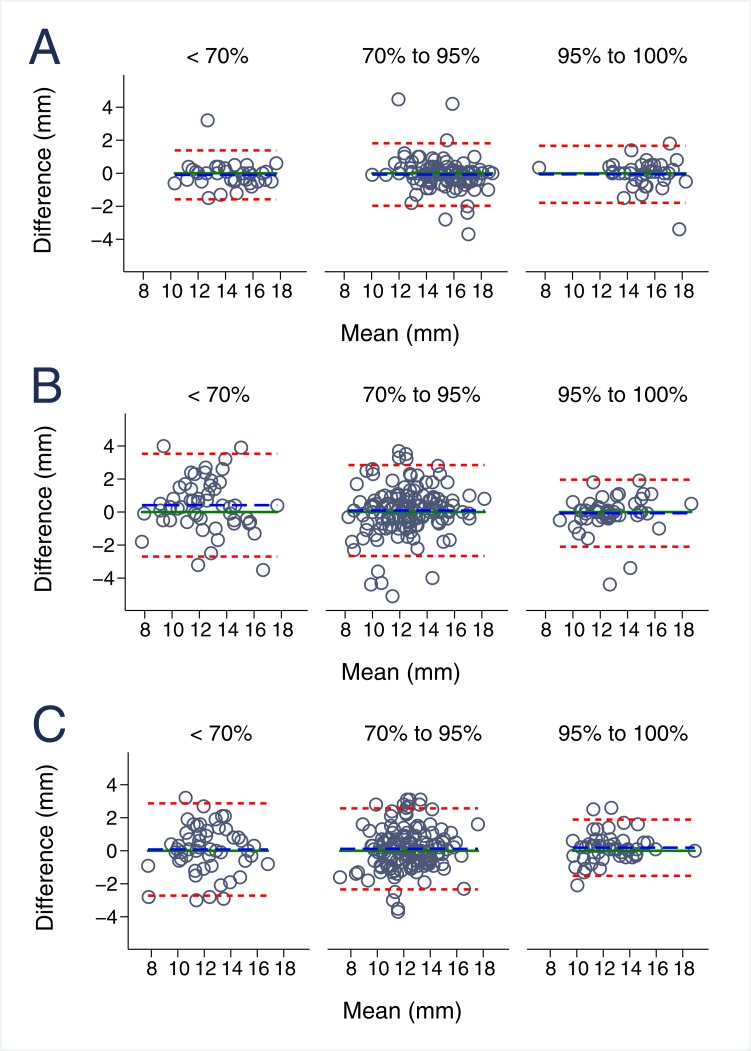
Interobserver reproducibility of MAPSE imaging in relation to echocardiographic image quality. **A.** Interobserver variability for m-mode derived MAPSE, **B.** Interobserver variability for B-Mode lateral derived MAPSE, **C.** Interobserver variability for B-Mode septal derived MAPSE.

**Fig 5:**
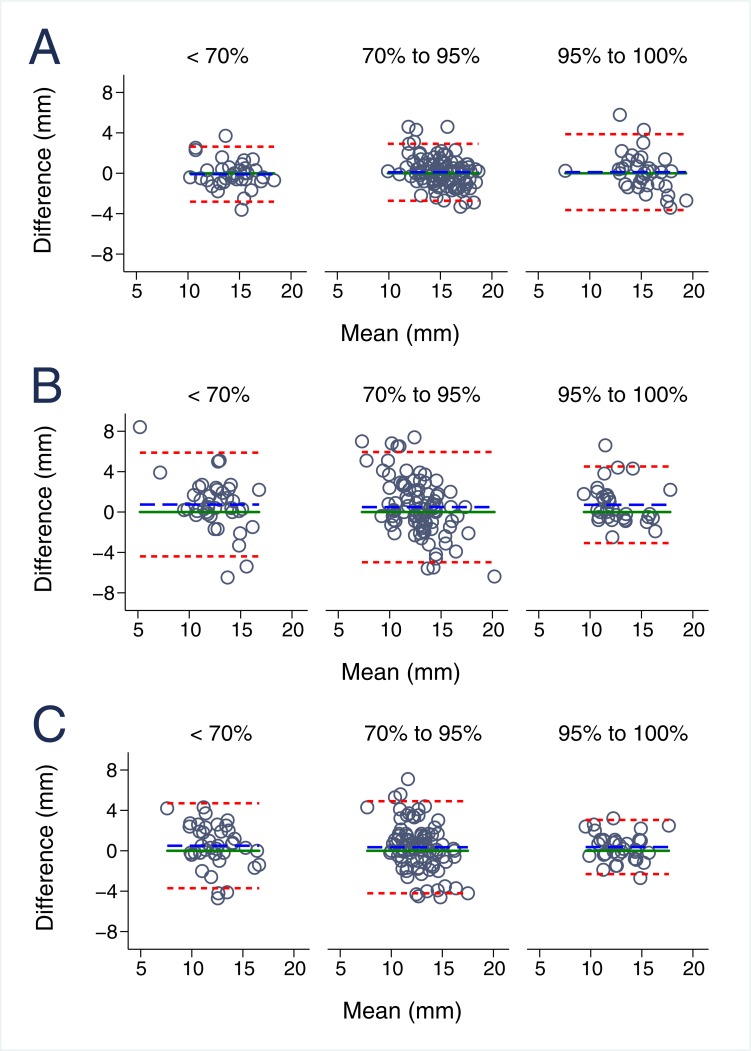
Intraobserver reproducibility of MAPSE imaging in relation to echocardiographic image quality. **A.** Intraobserver variability for m-mode derived MAPSE, **B.** Intraobserver variability for B-Mode lateral derived MAPSE, **C.** Intraobserver variability for B-Mode septal derived MAPSE.

The influence of echocardiographic image quality on MPASE variability is demonstrated in Table 3 and Fig 4 for interobserver and in Table 4 and Fig 5 for intraobserver comparisons. Inter- and intraobserver reproducibility for M-mode MAPSE did not depend on echocardiographic image quality (p>0.495 and p>0.235, respectively). Moreover, B-mode lateral MAPSE was significantly better reproducible with smaller bias (-0.07±1.04) and narrower limits of agreement in optimal image quality when compared to suboptimal echocardiographic images (0.42±1.59) both for inter- (p<0.001) and intraobserver measurements (p = 0.047). B-mode septal comparisons behaved equally with statistically significant higher agreement in optimal image quality for interobserver (p = 0.008) and a similar but statistically non-significant tendency in intraobserver measurements.

**Table 3:** Interobserver reproducibility of MAPSE measurements in relation to echocardiographic image quality. P-values were calculated with the Mann-Whitney-U-Test.

			Image quality	n	Mean	Sd	Median	Min-Max	rho*	95%-CI of the mean	Limits of agreement
**M-MODE LATERAL**											
	**Mean**	**1**	**< 70%**	36	-0.10	0.76	0.00	-1.50–3.20	0.93	-0.35; 0.16	-1.58; 1.39
	**difference**	**2**	**70% to 95%**	110	-0.08	0.97	0.00	-3.70–4.49	0.87	-0.26; 0.11	-1.97; 1.82
		**3**	**95% to 100%**	36	-0.06	0.88	0.00	-3.40–1.80	0.90	-0.36; 0.24	-1.79; 1.66
			**Total**	182	-0.08	0.91	0.00	-3.70–4.49	0.89	-0.21; 0.05	-1.85; 1.70
	**Absolute**	**1**	**< 70%**	36	0.47	0.59	0.40	0.00–3.20	2 vs. 1: p = 1.000		
	**difference**	**2**	**70% to 95%**	110	0.56	0.79	0.40	0.00–4.49	3 vs. 2: p = 0.495		
		**3**	**95% to 100%**	36	0.57	0.67	0.45	0.00–3.40	1 vs. 3: p = 0.463		
			**Total**	182	0.54	0.73	0.40	0.00–4.49			
**B-MODE LATERAL**											
	**Mean**	**1**	**< 70%**	52	0.42	1.59	0.35	-3.50–4.00	0.77	-0.03; 0.86	-2.70; 3.53
	**difference**	**2**	**70% to 95%**	143	0.09	1.41	0.20	-5.10–3.70	0.78	-0.14; 0.32	-2.66; 2.85
		**3**	**95% to 100%**	50	-0.07	1.04	0.00	-4.40–1.90	0.86	-0.36; 0.22	-2.10; 1.96
			**Total**	245	0.13	1.39	0.10	-5.10–4.00	0.79	-0.05; 0.30	-2.59; 2.84
	**Absolute**	**1**	**< 70%**	52	1.24	1.06	0.75	0.10–4.00	2 vs. 1: p = 0.088		
	**difference**	**2**	**70% to 95%**	143	0.99	1.00	0.70	0.00–5.10	3 vs. 2: p = 0.001		
		**3**	**95% to 100%**	50	0.60	0.84	0.30	0.00–4.40	1 vs. 3: p<0.001		
			**Total**	245	0.96	1.00	0.60	0.00–5.10			
**B-MODE SEPTAL**											
	**Mean**	**1**	**< 70%**	52	0.08	1.43	0.10	-3.00–3.20	0.76	-0.32; 0.48	-2.72; 2.87
	**difference**	**2**	**70% to 95%**	143	0.12	1.25	0.00	-3.70–3.10	0.79	-0.09; 0.32	-2.34; 2.57
		**3**	**95% to 100%**	50	0.19	0.87	0.10	-2.10–2.60	0.89	-0.06; 0.44	-1.52; 1.89
			**Total**	245	0.12	1.22	0.10	-3.70–3.20	0.80	-0.03; 0.28	-2.27; 2.52
	**Absolute**	**1**	**< 70%**	52	1.09	0.91	0.90	0.00–3.20	2 vs. 1: p = 0.274		
	**difference**	**2**	**70% to 95%**	143	0.93	0.84	0.70	0.00–3.70	3 vs. 2: p = 0.014		
		**3**	**95% to 100%**	50	0.62	0.63	0.40	0.00–2.60	1 vs. 3: p = 0.008		
			**Total**	245	0.90	0.83	0.60	0.00–3.70			

* Spearman's rank correlation coefficient

**Table 4:** Intraobserver reproducibility of MAPSE measurements in relation to echocardiographic image quality. P-values were calculated with the Mann-Whitney-U-Test.

			Image quality	n	Mean	Sd	Median	Min-Max	rho*	95%-CI of the mean	Limits of agreement
**M-MODE LATERAL**											
	**Mean**	**1**	**< 70%**	36	-0.10	0.76	0.00	-1.50–3.20	0.93	-0.35; 0.16	-1.58; 1.39
	**difference**	**2**	**70% to 95%**	110	-0.08	0.97	0.00	-3.70–4.49	0.87	-0.26; 0.11	-1.97; 1.82
		**3**	**95% to 100%**	36	-0.06	0.88	0.00	-3.40–1.80	0.90	-0.36; 0.24	-1.79; 1.66
			**Total**	182	-0.08	0.91	0.00	-3.70–4.49	0.89	-0.21; 0.05	-1.85; 1.70
	**Absolute**	**1**	**< 70%**	36	0.47	0.59	0.40	0.00–3.20	2 vs. 1: p = 0.643		
	**difference**	**2**	**70% to 95%**	110	0.56	0.79	0.40	0.00–4.49	3 vs. 2: p = 0.235		
		**3**	**95% to 100%**	36	0.57	0.67	0.45	0.00–3.40	1 vs. 3: p = 0.247		
			**Total**	182	0.54	0.73	0.40	0.00–4.49			
**B-MODE LATERAL**											
	**Mean**	**1**	**< 70%**	52	0.42	1.59	0.35	-3.50–4.00	0.77	-0.03; 0.86	-2.70; 3.53
	**difference**	**2**	**70% to 95%**	143	0.09	1.41	0.20	-5.10–3.70	0.78	-0.14; 0.32	-2.66; 2.85
		**3**	**95% to 100%**	50	-0.07	1.04	0.00	-4.40–1.90	0.86	-0.36; 0.22	-2.10; 1.96
			**Total**	245	0.13	1.39	0.10	-5.10–4.00	0.79	-0.05; 0.30	-2.59; 2.84
	**Absolute**	**1**	**< 70%**	52	1.24	1.06	0.75	0.10–4.00	2 vs. 1: p = 0.356		
	**difference**	**2**	**70% to 95%**	143	0.99	1.00	0.70	0.00–5.10	3 vs. 2: p = 0.047		
		**3**	**95% to 100%**	50	0.60	0.84	0.30	0.00–4.40	1 vs. 3: p = 0.362		
			**Total**	245	0.96	1.00	0.60	0.00–5.10			
**B-MODE SEPTAL**											
	**Mean**	**1**	**< 70%**	52	0.08	1.43	0.10	-3.00–3.20	0.76	-0.32; 0.48	-2.72; 2.87
	**difference**	**2**	**70% to 95%**	143	0.12	1.25	0.00	-3.70–3.10	0.79	-0.09; 0.32	-2.34; 2.57
		**3**	**95% to 100%**	50	0.19	0.87	0.10	-2.10–2.60	0.89	-0.06; 0.44	-1.52; 1.89
			**Total**	245	0.12	1.22	0.10	-3.70–3.20	0.80	-0.03; 0.28	-2.27; 2.52
	**Absolute**	**1**	**< 70%**	52	1.09	0.91	0.90	0.00–3.20	2 vs. 1: p = 0.896		
	**difference**	**2**	**70% to 95%**	143	0.93	0.84	0.70	0.00–3.70	3 vs. 2: p = 0.118		
		**3**	**95% to 100%**	50	0.62	0.63	0.40	0.00–2.60	1 vs. 3: p = 0.183		
			**Total**	245	0.90	0.83	0.60	0.00–3.70			

* Spearman's rank correlation coefficient

### The impact of echocardiographic image quality on MAPSE measurements

Interestingly, echocardiographic image quality significantly influenced M-mode MAPSE but not B-mode derived MAPSE (S2 Fig). Specifically, substandard quality images yielded significantly lower M-mode MAPSE values (14.3±2 mm) than near-optimal (15.2±1.9 mm, p<0.001) or optimal images (15.1±2.2 mm, p = 0.006). In B-mode derived septal and lateral MAPSE measurements differences in image quality did not result in significantly different MAPSE measurements (p>0.05).

## Discussion

In clinical echocardiography the most important information obtained is often the assessment of LV systolic function. While in hand of an experienced echocardiographer visual estimation (“eyeballing”) frequently serves as a sufficient approach, the evaluation of LV performance may require additional quantitative methods in technically difficult patients, i.e. children. MAPSE has been suggested as such a valid surrogate echocardiographic tool. In this study B-mode and M-mode derived MAPSE measurements in children featured excellent reproducibility with only minor bias. This is in agreement with echocardiography [30] and MRI [31] studies of mitral annular displacement measurements in adults. While MAPSE detection was convincingly proven useful and reliable in adults [6], this is the first study to analyze the methodological validity of MAPSE in children. Even though there is no specific pediatric data on reproducibility to compare our data with, the fact that MAPSE was shown to be an utterly sensitive tool to successfully detect even minor alterations of LV mechanics in several (sub-) clinical settings renders it likely to feature correspondingly strong variability. Firstly, MAPSE was decreased even in early stages of twin-to-twin transfusion syndrome before and after laser surgery [32]. Secondly, MAPSE has been reported not to be inferior to speckle tracking echocardiography derived global longitudinal peak strain for the determination of LV deterioration in aortic stenosis [33]. Recently, MAPSE was utilized as the functional reference to measure systolic LV longitudinal deformation in a study analyzing tortuosity of the coronary arteries [26]. Taken together, these examples indicate a degree of sensitivity, that is likely to be associated with the here reported reproducibility. Specifically, we found that both inter- and intraobserver reliability were significantly better for M-mode derived MAPSE. This is in accordance with a recent well-conducted study that also showed strong reproducibility of M-mode derived MAPSE in adult patients with various heart conditions [34]. However, the authors did not compare M-mode and B-mode MAPSE but speckle tracking and tissue-Doppler imaging, which showed the greatest degree of reproducibility. Similarly, in another study the reliability of fractional shortening measurements in children with dilated cardiomyopathy utilizing M-mode was superior to B-mode [35]. Nevertheless, even though statistically significant, the difference in reproducibility of M-mode and B-mode derived MAPSE in this study were ultimately still minor and, therefore, this difference should not be overestimated.

A major focus in the present study was the relevance of echocardiographic image quality for the reliability of MAPSE measurements. Interestingly, image quality did not significantly influence M-mode MAPSE reproducibility. In contrast, B-mode lateral MAPSE was significantly better reproducible in optimal image quality when compared to suboptimal echocardiographic images. While the overall effect of image quality in MAPSE assessment was small, the here described influences are to a certain extent against the previously proclaimed principle that MAPSE measurements are reliable irrespective of the degree of imaging quality. This phenomenon has been hypothesized to be due to the high echogenicity in the atrioventricular annulus [36]. In this sense Koestenberger and colleagues recently suggested that the determination of LV function using MAPSE might be useful for vulnerable infants for whom a prolonged examination is inappropriate and for neonates with suboptimal visualization of the endocardium [37]. In another study speckle tracking derived MAPSE measurements were shown to provide quick, easy, robust, and accurate estimates of EF irrespective of LV endocardial definition [38]. Moreover, in the present study poor quality images yielded lower M-mode MAPSE values than near-optimal or flawless images. However, these findings should be considered with caution and not be overinterpreted. Specifically, given the nature of the study design, causality cannot be explored. Furthermore, at the end of the day the biological relevance of the overall minor difference may be appreciated to be probably low. Further studies are needed to determine, whether image quality-based bias of MAPSE detection is a valid phenomenon and finally, whether it is ultimately likely to alter clinical decision making.

To evaluate the correspondence of MAPSE and other echocardiographic LV performance parameters, we carried out correlation analyses. In detail, we found that among others MAPSE correlated well with EF, longitudinal strain and strain rate. This is in accordance with findings from several studies reporting that, among various parameters, global longitudinal strain and EF were significantly associated with MAPSE [30, 39]. Moreover, the correlation of MAPSE and other functional LV assessment parameters does not seem to be limited to B-mode or M-mode derived MAPSE detection. Suzuki and colleagues utilized speckle tracking imaging to analyze MAPSE in different heart diseases and found a strong correlation of mitral annular displacement and EF, i.e. in patients with IHD and dilated cardiomyopathy [40]. Similarly, another study reported a significant correlation between mitral annular peak systolic velocity and MAPSE values in as many as 690 healthy children [41]. Finally, Matos and colleagues convincingly demonstrated, that MAPSE predicts EF even when performed by an untrained examiner [11]. The agreement of MAPSE and other quantitative echocardiographic LV performance parameters is assuring in that it underlines the functional consensus of these different methodological approaches, the validity of our findings and hence, ultimately, their diagnostic utility in everyday clinical medicine.

## Conclusion

MAPSE measurements showed excellent inter- and intraobserver reliability in children without structural heart disease. Furthermore, echocardiographic image quality had only minor effects on MAPSE detection. However, M-mode derived MAPSE values were slightly but statistically significantly lower in limited image quality. B-mode MAPSE was better reproducible in optimal image quality when compared to suboptimal echocardiographic images. In conclusion, MAPSE is a valuable echocardiographic tool for the assessment of LV function even in pediatric patients with suboptimal imaging conditions.

## Supporting information

S1 Text Correlation of MAPSE with conventional and novel echocardiographic parameters. (PDF)

S1 Table Correlation coefficients (r) of MAPSE imaging and other echocardiographic myocardial performance parameters (Spearman's rank correlation coefficient). (PDF)

S1 Fig Scatter plots demonstrating measurements of MAPSE and other echocardiographic myocardial performance parameters. (EPS)

S2 Fig MAPSE measurements according to echocardiographic image quality. (EPS)

S1 Source data Complete raw data set. (XLSX)
