# Supplementary material for: Intraobserver and interobserver reproducibility of M-mode and B-mode acquired mitral annular plane systolic excursion (MAPSE) and its dependency on echocardiographic image quality in children
Source: PLoS One. 2018 May 10;13(5):e0196614. doi: 10.1371/journal.pone.0196614 (PMC5944962; doi:10.1371/journal.pone.0196614)
Supplement: S1 Text — (PDF) [file pone.0196614.s001.pdf]

S1 text. Correlation of MAPSE with conventional and novel echocardiographic parameters.

Correlations of MAPSE and other echocardiographic myocardial performance parameters are presented in supplemental table S1 and supplemental figure S1. B-mode septal and lateral MAPSE measurements correlated better (rho=0.74; $p<0.001$) than B-mode (septal and lateral) and M-mode derived MAPSE values (rho=0.32 and rho=0.19, respectively; $p<0.001$). B-mode and M-mode MAPSE correlated with EF (rho=0.52 and rho=0.28, respectively; $p<0.001$) and LV mass (rho=0.29 and rho=0.23, respectively; $p\leq0.001$). Global peak LV longitudinal strain showed significant positive correlation with B-mode septal (rho=0.26; p=0.001) and lateral (rho=0.21; p=0.006) as well as with M-mode derived MAPSE (rho=0.19; p=0.023). Global peak LV longitudinal strain rate correlated significantly with B-mode (rho=0.32; $p<0.001$) but not with M-mode derived MAPSE (p=0.160).
