# Supplementary material for: Intraobserver and interobserver reproducibility of M-mode and B-mode acquired mitral annular plane systolic excursion (MAPSE) and its dependency on echocardiographic image quality in children
Source: PLoS One. 2018 May 10;13(5):e0196614. doi: 10.1371/journal.pone.0196614 (PMC5944962; doi:10.1371/journal.pone.0196614)
Supplement: S1 Table — (PDF) [file pone.0196614.s002.pdf]

S1 table. Correlation coefficients (r) of MAPSE imaging and other echocardiographic myocardial performance parameters (Spearman's rank correlation coefficient)

	M-Mode derived MAPSE			B-mode lateral derived MAPSE			B-mode septal derived MAPSE		
	**n**	**rho**	**p-value**	**n**	**rho**	**p-value**	**n**	**rho**	**p-value**
**M-mode derived MAPSE**	-	-	-	187	0.32	<0.001	187	0.19	0.009
**B-mode lateral derived MAPSE**	187	0.32	<0.001	-	-	-	251	0.74	<0.001
**B-mode septal derived MAPSE**	187	0.19	0.009	251	0.74	<0.001	-	-	-
**Left ventricular ejection fraction**	210	0.28	<0.001	250	0.44	<0.001	250	0.52	<0.001
**Stroke volume**	192	0.30	<0.001	215	0.38	<0.001	215	0.39	<0.001
**Left ventricular mass**	190	0.23	0.001	213	0.29	<0.001	213	0.29	<0.001
**E/A**	182	0.12	0.095	207	0.04	0.534	207	0.09	0.199
**E/E'**	182	-0.16	0.032	206	-0.12	0.090	206	-0.22	0.001
**Global circumferential strain**	138	0.09	0.290	168	0.06	0.463	168	0.13	0.098
**Global longitudinal strain**	138	0.19	0.023	166	0.21	0.006	166	0.26	0.001
**Global circumferential strain rate**	139	-0.24	0.005	171	-0.25	0.001	171	-0.15	0.047
**Global longitudinal strain rate**	138	0.12	0.160	169	0.28	<0.001	169	0.32	<0.001
**MAPSE / length**	210	0.65	<0.001	187	0.27	<0.001	187	0.23	0.002
